# Electric dipole of InN/InGaN quantum dots and holes and giant surface photovoltage directly measured by Kelvin probe force microscopy

**DOI:** 10.1038/s41598-020-62820-3

**Published:** 2020-04-03

**Authors:** Yinping Qian, Peng Wang, Lujia Rao, Changkun Song, Hongjie Yin, Xingyu Wang, Guofu Zhou, Richard Nötzel

**Affiliations:** 1 Guangdong Provincial Key Laboratory of Optical Information Materials and Technology, South China Academy of Advanced Optoelectronics, South China Normal University, Guangzhou, 510006 P. R. China; 2 National Center for International Research on Green Optoelectronics, South China Normal University, Guangzhou, 510006 P. R. China; 3 Academy of Shenzhen Guohua Optoelectronics, Shenzhen, 518110 P. R. China

**Keywords:** Materials science, Nanoscience and technology

## Abstract

We directly measure the electric dipole of InN quantum dots (QDs) grown on In-rich InGaN layers by Kelvin probe force microscopy. This significantly advances the understanding of the superior catalytic performance of InN/InGaN QDs in ion- and biosensing and in photoelectrochemical hydrogen generation by water splitting and the understanding of the important third-generation InGaN semiconductor surface in general. The positive surface photovoltage (SPV) gives an outward QD dipole with dipole potential of the order of 150 mV, in agreement with previous calculations. After HCl-etching, to complement the determination of the electric dipole, a giant negative SPV of −2.4 V, significantly larger than the InGaN bandgap energy, is discovered. This giant SPV is assigned to a large inward electric dipole, associated with the appearance of holes, matching the original QD lateral size and density. Such surprising result points towards unique photovoltaic effects and photosensitivity.

## Introduction

Beyond the established applications of III-nitride semiconductors for blue lasers, solid state lighting and high-power amplifiers, the great potential of InGaN for electrochemical devices has been widely recognized recently. This is due to the intrinsic materials properties of InGaN, most relevant, the wide direct bandgap tunability upon In content from the ultraviolet to the near-infrared spectral region, the huge near bandgap absorption coefficient, one order of magnitude larger than that of GaAs, the large carrier mobility, the non toxicity and bio-compatibility. Moreover, InGaN exhibits unique surface properties, governing the electrochemical activity. For the c-plane of InGaN there exists a transition from negatively to positively charged surface states above about 40% In content, reaching a density of 2 × 10^13^ cm^−2^ for InN. The associated transition from surface electron depletion to surface electron accumulation, i.e., from upward surface energy band bending to downward surface energy band bending, i.e., from positive to negative surface photovoltage (SPV) has been directly revealed by electrochemical capacitance-voltage (C-V) measurements, X-ray photoelectron spectroscopy (XPS), and Kelvin probe force microscopy (KPFM)^1–4^. The positively charged surface states are highly active to attract anions and electrons to catalytically enhance oxidation reactions. Key applications include electrochemical ion- and biosensors^5–7^ and photoelectrochemical solar hydrogen generation by water splitting^8–11^.

In both application areas, InN/InGaN quantum dots (QDs) show superior performance^12–14^. Super-Nernstian sensitivity is observed in potentiometric sensing and the efficiency of hydrogen generation by water splitting is enhanced by almost a factor of three compared to that for a bare InGaN layer. This has been explained by the existence of a large electric dipole associated with the InN QDs in addition to the pure surface charge effect. For the QDs, the high density of positively charged surface states is not fully compensated because not sufficient electrons can enter the QDs due to the zero-dimensional electronic states obeying the Pauli exclusion principle. The electrons are partly expelled into the InGaN layer underneath the QDs and form a large electric dipole together with the uncompensated, positively charged surface states, with the electric dipole moment pointing outwards. The strength of the electric dipole non-trivially depends on the QD size and shape, establishing an artificial, tunable electrocatalyst^15^.

Here we report the direct measurement of the electric dipole of InN/InGaN QDs by KPFM^16–18^. Sample growth is performed by plasma-assisted molecular beam epitaxy (PA-MBE) on metalorganic vapor phase epitaxy (MOVPE) grown GaN/sapphire templates. Exemplary, 1.2-monolayer (ML)-InN QDs grown on an In-rich In_0.45_Ga_0.55_N layer are discussed. For comparison, a 0.8-ML-InN/In_0.45_Ga_0.55_N structure with InN amount below the onset of QD formation and a 1.5-ML-InN/In_0.75_Ga_0.25_N QD structure are investigated. For the InGaN layer with 75% In content, the InN deposition amount is increased to compensate for the smaller lattice mismatch for QD formation. The number of InN MLs is determined from calibration samples which are InGaN layers with different In content deduced from X-ray diffraction (XRD) and thickness deduced from scanning electron microscopy (SEM). In the KPFM measurements we determine the contact potential difference (CPD), which is the difference between the metal tip- and sample work functions and the SPV, which is the difference of the CPD under illumination and the CPD in the dark. A strong outward QD electric dipole with a dipole potential not smaller than 150 mV is deduced. The importance of the two-dimensional wetting layer (WL) underneath the QDs to realize a stable, molecule-like, permanent dipole is unraveled. Etching in HCl is performed to exclude influences of the surface morphology. Surprisingly, the CPD and SPV indicate a strong inward electric dipole after etching, which is associated with the appearance of holes matching the original QD lateral size and density. A giant negative SPV of −2.4 V is measured, much larger in magnitude than the InGaN bandgap energy. Respective energy band diagrams are given at the end for full clarification.

## Results

### As-grown

**Figure 1:**
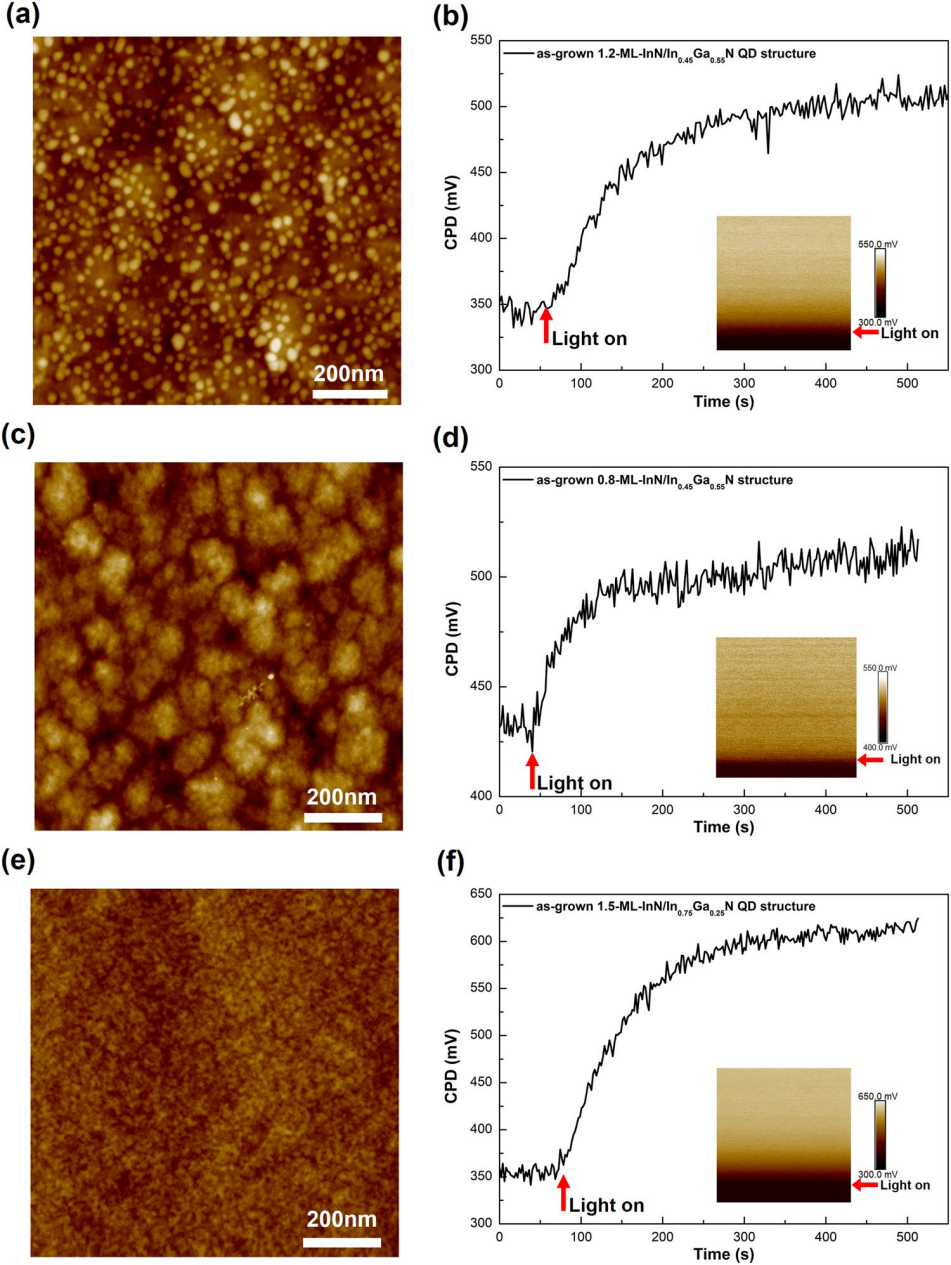
As-grown InN/InGaN structures. AFM height images and CPD as a function of time for the as-grown (**a**,**b**) 1.2-ML-InN/In_0.45_Ga_0.55_N QD structure, (**c**,**d**) 0.8-ML-InN/In_0.45_Ga_0.55_N structure and (**e**,**f**) 1.5-ML-InN/In_0.75_Ga_0.25_N QD structure. Insets in (**b**,**d**,**f**): KPFM CPD images. The arrows indicate the switching on of the light. The scan field is 1 × 1 µm^2^ and the full height contrast is 10 nm in all AFM images.

Figure 1 shows the atomic force microscopy (AFM) height images and CPD as a function of time for the as-grown (a, b) 1.2-ML-InN/In_0.45_Ga_0.55_N QD structure, (c, d) 0.8-ML-InN/In_0.45_Ga_0.55_N structure and (e, f) 1.5-ML-InN/In_0.75_Ga_0.25_N QD structure. The insets in the CPD traces in (b, d, f) show the corresponding KPFM CPD images. The arrows indicate the switching on of the light. The AFM root-mean-square (rms) roughness, the dark CPD-, light CPD- and SPV values of the samples are listed in Table 1. The SEM images, XRD- and photoluminescence (PL) spectra for sample characterization are shown in Supplementary Figs. S1, S2 and S3, respectively. For the 1.2-ML-InN/In_0.45_Ga_0.55_N structure, QDs are well developed. The AFM image is typical for such self-assembled epitaxial QDs grown in the Stranski-Krastanov mode. Evaluation of AFM line scans over 250 QDs reveals the average height of 5 nm, average base width of 30 nm (overestimated in AFM due to tip convolution) and a density of 8 × 10^10^ cm^−2^. For the 0.8-ML-InN/In_0.45_Ga_0.55_N structure an undulated surface is seen, showing that 0.8 ML InN is below the critical thickness for QD formation. For the 1.5-ML-InN/In_0.75_Ga_0.25_N structure, although the InN deposition amount is increased, the QDs are less developed and much smaller due to the lower growth temperature and reduced lattice mismatch between InN and InGaN. The deposition amount is not further increased to avoid QD coalescence. High-resolution cross-sectional transmission electron microscopy (TEM) images of such QDs have been reported in ref. ^19^. The QD shown exhibits a height of 3 nm and base width of 10 nm. In the QD ensemble, the height varies between 2 and 4 nm and the base width between 10 and 40 nm. Differences in size, shape and density depend on the inherent statistic distributions for such QDs and the precise growth conditions. Therefore, only qualitatively different structures, InN QDs, modulated InN layer and different In composition of the InGaN layer are compared. The CPD increases upon illumination for all samples, indicating a positive SPV. The QDs, which uniformly cover the surface, cannot be individually resolved in the KPFM CPD images due to the 100 nm spatial resolution, leading to a uniform contrast.

**Table 1:** Process and KPFM results.

	Process	rms (nm)	CPD dark (V)	CPD light (V)	SPV (V)
1.2-ML-InN/In_0.45_Ga_0.55_N QD structure	as-grown	2.4	0.35	0.50	0.15
	HCl-etched	0.81	−1.0	−3.4	−2.4
0.8-ML-InN/In_0.45_Ga_0.55_N structure	as-grown	2.0	0.43	0.51	0.075
	HCl-etched	2.4	0.74	0.40	−0.34
1.5-ML-InN/In_0.75_Ga_0.25_N QD structure	as-grown	0.44	0.35	0.6	0.25
	HCl-etched	0.60	−0.40	−2.1	−1.7

AFM root-mean-square (rms) roughness, contact potential difference (CPD) in the dark and light, and surface photovoltage (SPV) for the as-grown and HCl-etched 1.2-ML-InN/In_0.45_Ga_0.55_N QD structure, 0.8-ML-InN/In_0.45_Ga_0.55_N structure and 1.5-ML-InN/In_0.75_Ga_0.25_N QD structure, as indicated.

### HCl-etched

**Figure 2:**
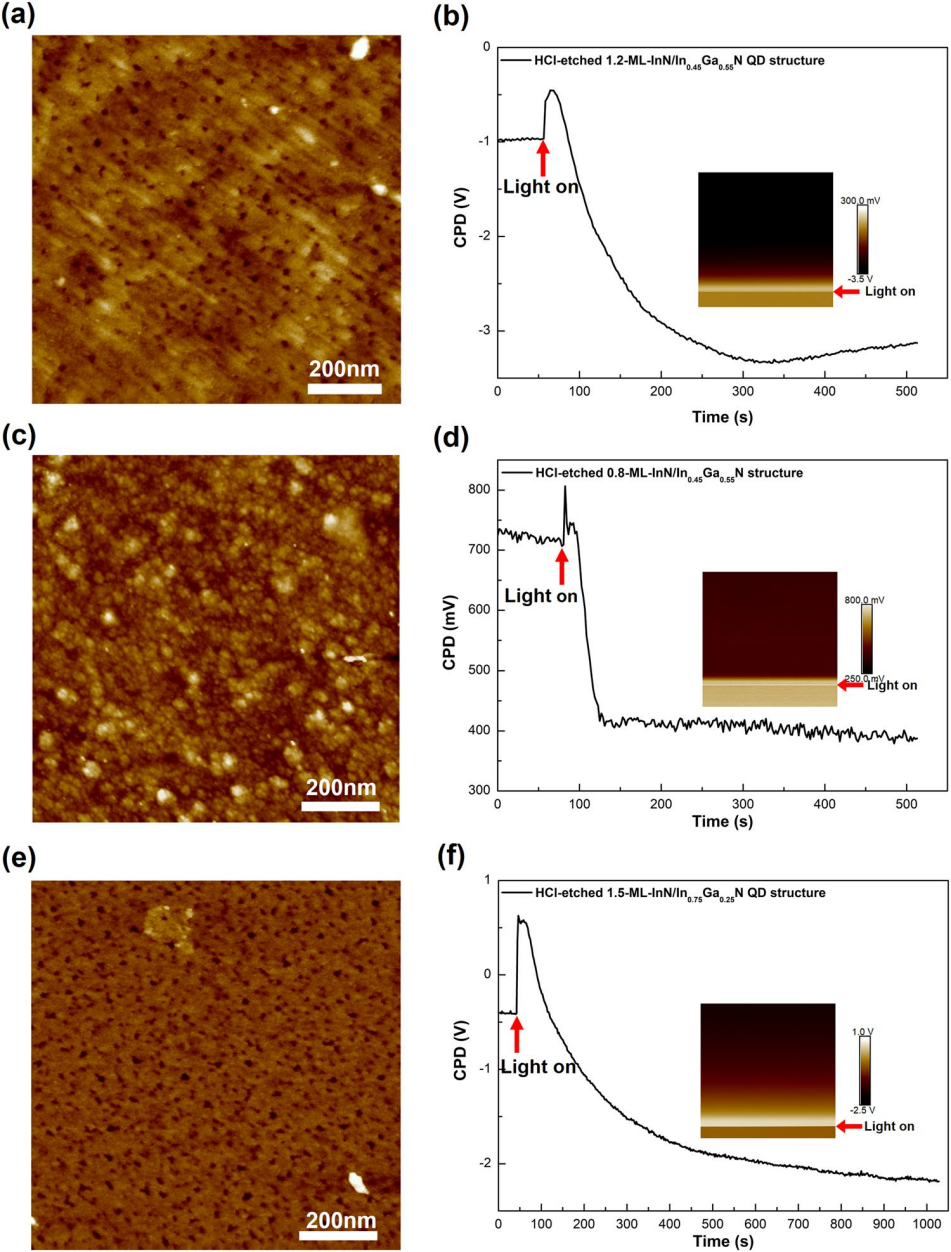
HCl-etched InN/InGaN structures. AFM height images and CPD as a function of time after HCl-etching for the (**a**,**b**) 1.2-ML-InN/In_0.45_Ga_0.55_N QD structure, (**c**,**d**) 0.8-ML-InN/In_0.45_Ga_0.55_N structure and (**e**,**f**) 1.5-ML-InN/In_0.75_Ga_0.25_N QD structure. Insets in (**b**,**d**,**f**): KPFM CPD images. The arrows indicate the switching on of the light. The scan field is 1 × 1 µm^2^ and the full height contrast is10 nm in all AFM images.

Figure 2 shows the AFM height images and CPD as a function of time after HCl-etching for the (a, b) 1.2-ML-InN/In_0.45_Ga_0.55_N QD structure, (c, d) 0.8-ML-InN/In_0.45_Ga_0.55_N structure and (e, f) 1.5-ML-InN/In_0.75_Ga_0.25_N QD structure. The insets in the CPD traces in (b, d, f) show the corresponding KPFM CPD images. The arrows indicate the switching on of the light. The AFM rms roughness, the dark CPD-, light CPD- and SPV values of the samples are also listed in Table 1. The SEM images are shown in Supplementary Fig. S4.

The surface morphology observed in the AFM images in Fig. 2 reveals the removal of the c-plane InN QDs and InN surface layers upon HCl-etching. After etching the QD layers, a flat island surface morphology is observed. In particular for the 1.2-ML-InN/In_0.45_Ga_0.55_N QD structure with well developed QDs, ML-high islands are resolved in AFM line scans and the rms roughness is clearly reduced after HCl etching. Unexpectedly, together with this flattened surface, holes appear. The lateral size and density of the holes match the lateral size and density of the original QDs, most obvious for the well-developed QDs in Fig. 1(a). Therefore, the etching of the holes appears to be triggered by the QDs, likely due to strain and/or defects at the original InN/InGaN interface underneath the QDs. This is further put forward because no distinct holes are visible after etching the 0.8-ML-InN/In_0.45_Ga_0.55_N structure having no QDs. The surface only becomes more densely rippled without much change of the rms roughness. The holes after QD etching are not that deep, approximately 3–4 nm, deduced from AFM line scans over 250 holes, though the AFM tip may not reach the bottom of the holes. The CPD exhibits a quick increase followed by a pronounced decrease upon illumination for all samples, indicating an overall negative SPV. The holes, which uniformly cover the surface, cannot be individually resolved in the KPFM CPD images due to the 100 nm spatial resolution, leading to a uniform contrast.

## Discussion

### QD dipole

In the KPFM measurements, for all the as-grown samples in Fig. 1, both, the dark CPD, which is used in all further discussions, and the SPV are positive. This opposes the behavior expected due to the positively charged surface states^4^. Especially, the SPV should be negative and actually become more negative with increasing InN amount, when more positive charge is added. Therefore, in addition to the positive surface charge, another, counteracting property is introduced by the QDs. This identifies the proposed outward electric dipole of the QDs. An electric dipole potential not smaller than 150 mV is indicated, taking the SPV of the 1.2-ML-InN/In_0.45_Ga_0.55_N QD structure as conservative lower limit. This is justified by the fact that the In content of 45% is very close to the transition point from negatively to positively charged surface states, i.e., very close to flat-band condition. The deduced value of the QD dipole potential is in agreement with calculations based on the QD size and shape, number of confined electron states and number of positively charged surface states. An analytical model, which is described in detail in ref. ^20^, treats the truncated pyramid shaped QD as two oppositely charged circular disks with radius r and distance h. This gives an electric dipole potential, evaluated along the axis through the center of the disks,

1 $${V}_{dipole}=\frac{\sigma }{2{\varepsilon }_{0}{\varepsilon }_{r}}(r+h-\sqrt{{r}^{2}+{h}^{2}}),$$

around 200 mV. σ is the uncompensated positive surface charge density assuming three to four bound electron states and a density of positively charged surface states of 2 × 10^13^ cm^−2^, r is the QD radius taken as 10–15 nm, h is the QD height taken as 3–5 nm, ε_0_ is the vacuum permittivity and ε_r_ is the relative permittivity of InN of 11–15. Considering the uncertainties in the precise QD size, shape, composition, strain distribution, surface charge density on the various exposed planes, relative permittivity, energy band alignment, band offsets, effective electron mass, etc., we believe such a simplified model is reasonable to grab the essence and provide an approximate quantification. For the 0.8-ML-InN/In_0.45_Ga_0.55_N structure this means an electric dipole also for InN amounts below QD formation which may well be present for not perfectly planar layers. This dipole is, however, weaker, which is reflected in the smaller positive SPV compared to that for the QDs.

The CPD and SPV measured by KPFM are due to the combined effect of surface charge and electric dipole. The electric dipole is formed by separating the surface electron accumulation layer from the positively charged surface states due to quantum repulsion. A sufficiently large QD electric dipole eventually overcompensates the positive surface charge. It attracts holes to the surface, meaning, it turns the downward energy band bending to an upward energy band bending and the SPV from negative- to positive values. Therefore, the positive SPV observed experimentally implies that the QDs act as stable, permanent electric dipoles on the InGaN surface, much like molecules with a permanent dipole moment^21–23^. For very high In content, the downward energy band bending gets that steep due to very high density of positively charged surface states that tunneling occurs. The upward energy band bending then refers to the bulk for any QD electric dipole to attract holes to the surface leading to positive SPV.

A stable, permanent QD electric dipole requires that the electrons expelled from the QDs stay confined close to the QDs. In the present QD system this confinement is provided by the two-dimensional InN WL underneath the QDs, which is formed for the InN QDs grown in the Stranski-Krastanov mode. The presence of the two-dimensional WL was clearly revealed by cross-sectional TEM^19^. Therefore, we refine our model, which reads now: “Due to the Pauli exclusion principle, not sufficient electrons can enter the QDs to fully compensate the positively charged surface states. Electrons are partly expelled from the QDs and confined in the two-dimensional WL underneath to form a stable, molecule-like electric dipole together with the uncompensated, positively charged surface states.”

Regarding the dynamic properties, the process to reach steady state conditions of the CPD after switching on the light is relatively slow. Typical time constants to reach 90% of the steady state value are 150 s. This is probably because the process involves holes reaching the InGaN surface, encountering the QDs, relax through the WL to the QD ground state and recombine with electrons in the QDs to leave an excess number of fixed positively charged surface states. Such ‘slow’ processes involving charge transfer of photogenerated carriers between the bulk semiconductor and surface states or, here, surface dipoles are to be distinguished from ‘fast’ processes involving only charge accumulation of photogenerated carriers at the semiconductor surface to screen the surface states^24^.

So far we have not considered the influence of the surface morphology on the measured CPD and SPV values, which are an average over the values for the different exposed facets/planes on non-planar surfaces. This neglect of the surface morphology is well justified by the very planar surfaces of all samples with small rms roughness. To independently exclude effects due to the surface morphology, the etching experiments in HCl aqueous solution are performed. This is motivated because etching is found to selectively remove the c-plane InN QD- and InN surface layers. Hence, etching is expected to remove the outward electric dipole, leaving positively charged surface states alone. This should not influence the CPD too much. The CPD likely increases as the positive surface charge alone, if remaining from the InN QD- or InN surface layers, rather has a larger influence than the outward electric dipole. The SPV, on the other hand, must turn from positive- to negative values. In contrast, for non-planar surfaces, in particular for columnar structures with exposed m-plane sidewalls, the SPV introduced by the sidewalls is always positive due to the negatively charged surface states of m-planes for the entire In composition range^3^. If there is a metallic In ad-layer on the m-plane sidewalls before etching, which may cause electron surface accumulation and negative SPV^3^, the SPV turns positive upon etching when the In ad-layer is removed. In the experiments, shown in Fig. 2, we always observe a reduction of the SPV to negative values upon etching. Therefore, the positive SPV in the presence of the c-plane InN QDs and InN layers before etching originates from the outward electric dipole of the QDs and InN layers and not from any surface morphology, e.g., from exposed m-planes with positive SPV.

### Hole dipole

In more detail, the KPFM measurements after HCl-etching of the InN/InGaN QD structures, shown in Fig. 2(b,f), reveal a change for both, the CPD and the SPV from moderate positive values to large negative values. The CPD of the HCL-etched 0.8-ML-InN/In_0.45_Ga_0.55_N structure without QDs, shown in Fig. 2(d) stays positive, moderately increases. The SPV changes from moderate positive- to moderate negative values. This is the expected behavior when the outward electric dipole due to the c-plane InN layer is removed and the positive surface charge effect takes over. The SPV of −0.34 V, however, is too negative for a bare InGaN layer with 45% In content^4^, close to flat-band condition, and the CPD of 0.74 V is too large. Our as-grown, bare In_0.45_Ga_0.55_N layer has a SPV of −0.070 V and a CPD of 0.55 V, shown in Supplementary Fig. S5 together with the AFM image. However, also for such as-grown InGaN layers, InN surface layers may form due to In segregation, affecting the SPV and CPD values, but HCl etching does not show significant changes. Regardless, after etching the intentionally grown InN layer, a large amount of positive charge is clearly left on the In_0.45_Ga_0.55_N surface.

The behavior of the HCl-etched InN/InGaN QD structures cannot be explained by positively charged surface states alone. When both, the CPD and SPV become largely negative, there must be an inward electric dipole after etching. This inward electric dipole is associated with the appearance of the holes, being the only marked structural difference. To offer an explanation, the holes implicate the absence of positive surface charge at the top, with positive surface charge around, while the bottom surface of the holes is positively charged. This positive surface charge at the bottom together with the negative charge contrast at the top, with respect to the surrounding positive charge of the unetched surface, establish the inward electric dipole.

The SPV amounts to −2.4 and −1.7 V for the two samples with In content x of the InGaN layer of 0.45 and 0.75. The corresponding bandgap energies E_g_ of 1.7 and 1.2 eV are estimated from the PL spectra shown in Supplementary Fig. S3. The negative SPV values are markedly larger in magnitude than the InGaN bandgap energies. Such large negative SPV values are understood by taking into account the relative permittivity of vacuum of 1 for the holes, instead of 11–15 for the InN QDs. Therefore, the dipole potential of the holes, which is inversely proportional to the relative permittivity, is easily increased by more than an order of magnitude for similar geometrical size of the holes and QDs. The negatively charged m-plane hole sidewalls also contribute to increase the hole dipole potential.

Another important aspect of the inward electric dipole is suppressed surface recombination due to the related large work function. This is confirmed by the PL intensity, which is significantly larger, almost doubled for the etched InN/InGaN QD structures compared to the as-grown ones, shown in Supplementary Fig. S6 for the 1.2-ML-InAs/In_0.45_Ga_0.55_N QD structure.

Regarding dynamic properties, the process for the etched InN/InGaN QD structures to reach steady state conditions of the CPD is relatively slow. This might be due to hindered movement of electrons towards the surface by the etched holes in addition to electron redistribution involving surface states. There is also a significant initial increase of the CPD. This is attributed to a separate, lateral movement of photogenerated holes towards the m-plane etched hole sidewalls with upward band bending. This initial CPD increase is a fast process, light-on-off CPD traces are shown in Supplementary Fig. S7, involving photogenerated hole accumulation at the etched hole sidewalls, screening the m-plane, negatively charged surface states, without slower charge redistribution among surface states^24^. For the etched 0.8-ML-InN/In_0.45_Ga_0.55_N structure, the response to reach steady state conditions of the CPD is intermediately fast. Electron redistribution involves only surface states. There is also a weak initial increase of the CPD, probably due to the ripple sidewalls.

### Other impacts on surface charge and dipole

In general, removal of a thin native oxide layer on the uncapped InN QDs upon HCl etching can also change the charge state. However, we observe complete removal of the QDs and the appearance of holes. Oxide removal might increase the positive charge for the clean surface to reduce the SPV. But a pure surface charge effect cannot explain the magnitude of the negative CPD and SPV and the related inward electric dipole. Moreover, rapid re-oxidation occurs and we observe similar results when the samples are exposed to air for an extended period of time.

Finally, it should be noted, that the lattice polarity of InGaN is also an origin of an electric dipole. MBE-grown InGaN layers on MOVPE-grown GaN/sapphire templates, investigated here, are Ga-polar^25^ with inward electric dipole^26^. This is opposite in direction to the measured outward electric dipole for the as-grown structures. Thus, a lattice polarity induced electric dipole/polarization charge might only reduce the measured electric dipole, which can only be assigned to the positively charged surface states and negatively charged surface electron accumulation layer. Moreover, MBE-grown InGaN layers on Si substrates are N-polar with outward electric dipole. For both substrates, the experimental results are very similar^12,13^, indicating minor influence of the lattice polarity induced electric dipole. After etching, the lattice polarity induced electric dipole and the measured electric dipole both point inwards. But the values of the negative CPD and SPV are too large to be accounted for by lattice polarity and lattice polarity also contradicts the results for as-grown, bare InGaN layers^4^. A bulk lattice polarity induced electric dipole/polarization charge is probably easily (internally) screened^27,28^ at the surface unlike a charged surface states related electric dipole. Therefore, we conclude, that both the outward and inward measured electric dipoles are governed by the respective charge distributions of the QDs and holes.

### Energy band diagrams

**Figure 3:**
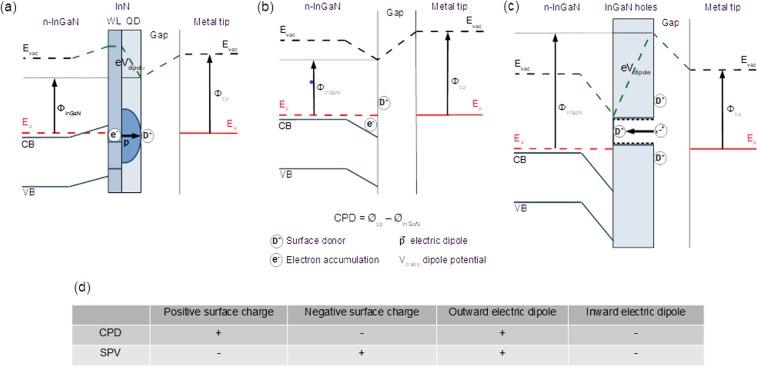
Band diagrams and CPD/SPV trends. Energy band diagrams in the dark together with sketches of the respective structures and charge distributions for the c-plane of an In-rich InGaN layer with (**a**) outward surface electric dipole, (**b**) positive surface charge alone and (**c**) inward surface electric dipole. The layers are in equilibrium with the metal tip. ϕ_tip_ and ϕ_InGaN_ are the tip- and sample work functions. V_dipole_ is the dipole potential. (**d**) Trends of the CPD and SPV for positive surface charge, negative surface charge, outward electric dipole and inward electric dipole. + indicates trend towards increasing, positive values and − indicates trend towards decreasing, negative CPD/SPV values for increase of the respective charge/dipole magnitude.

For final clarification, Fig. 3 shows energy band diagrams in the dark together with sketches of the respective structures and charge distributions for the c-plane of an In-rich InGaN layer (a) with outward surface electric dipole due to the InN QDs or non-planar InN layer with positively charged surface donors and expelled electrons to the WL (the SPV becomes positive for sufficiently strong electric dipole), (b) with positive surface charge alone when the outward surface electric dipole is removed after etching the non-planar InN layer (the CPD does not change much and the SPV decreases from positive- to negative values) and (c) with inward surface electric dipole due to the etched holes with positive surface charge at the bottom, missing positive surface charge at the top and negatively charged sidewalls (both, the CPD and SPV decrease to negative values). The In-rich InGaN layer is n-type degenerate due to a high density of defects acting as donors. The Table in (d) summarizes the respective trends of the CPD and SPV.

## Conclusions

To conclude, we have reported the direct measurement of the outward electric dipole associated with InN QDs grown on In-rich In_0.45_Ga_0.55_N layers by KPFM. For comparison, an InN/In_0.45_Ga_0.55_N structure with InN amount below the onset of QD formation and an InN/In_0.75_Ga_0.25_N QD structure were investigated. The CPD and SPV were positive for all InN/InGaN structures. This evidenced the strong outward QD electric dipole with a dipole potential not smaller than 150 mV. The importance of the wetting layer underneath the QDs was understood to realize a stable, molecule-like, permanent dipole. For the InN layer it followed that an electric dipole, though weaker, was also formed for an InN amount below QD formation. Etching in HCl was performed to independently assess the electric dipole and to exclude influences due to the surface morphology. For the InN/InGaN structure with InN amount below QD formation, the CPD stayed positive while the SPV became negative, revealing removal of the electric dipole and sole positive surface charge effect. In contrast, the CPD and SPV for the InN/InGaN QD structures both became highly negative. This unexpected behavior implied an inward electric dipole which was attributed to holes found after etching, matching the original QD lateral size and density. The SPV reached −2.4 V, much larger in magnitude than the InGaN bandgap energy. Together with the large negative SPV, a smaller, positive SPV was observed, associated with the hole sidewalls. These results point towards unique photovoltaic effects and photosensitivity.

## Methods

### Materials growth and processing

The InN/InGaN samples were grown by plasma-assisted molecular beam epitaxy (PA-MBE) on commercial metalorganic vapor-phase epitaxy (MOVPE) grown GaN-on-sapphire substrates. Active N was supplied by a radio-frequency (RF) plasma source^29^. The substrates were degassed for 30 min at 300 °C in the MBE load chamber before being transferred to the MBE growth chamber. The growth temperature for InGaN with an In content of 45% was 520 °C, close to the InGaN decomposition temperature and the growth rate was 190 nm/hour. The total InGaN layer thickness was 125 nm. The active N flux was close to stoichiometric, slightly N-rich growth conditions with 220 W/1.2 standard cubic centimeter per minute (sccm) plasma source settings. For InN growth the temperature was reduced to 450 °C. The InN amount was 1.2 ML and 0.8 ML. For comparison, a 1.5-ML-InN/125-nm-In_0.75_Ga_0.25_N QD structure was grown at 400 °C with a growth rate of 200 nm/hour and 265 W/1.7 sccm RF plasma source settings. Etching was performed in 15% HCl aqueous solution with ultrasonic stirring for 3 minutes.

### Materials assessment

The surface morphology of the samples was examined by atomic force microscopy (AFM) and scanning-electron microscopy (SEM), also in cross-sectional view. The In content of the InGaN layers was determined by X-ray diffraction (XRD). The energy bandgap was estimated from photoluminescence (PL) measurements at room temperature. The KPFM measurements were performed using a Pt-Ir coated silicon tip. The spatial resolution was about 100 nm. Therefore, the QDs and holes could not be resolved individually in the KPFM measurements. Before the measurements, the samples were stored for one day in the dark to reset any light induced charging processes. Illumination was by a 3 W Hg discharge lamp with power density of 3 mW/cm^2^ for above bandgap excitation.

## Supplementary information

Supplementary Information.

## Data Availability

All data needed to evaluate the conclusions in the paper are present in the paper and/or the Supplementary Materials. The data that support the findings in this study are available upon request from the corresponding author.
