# Supplementary material for: Electric dipole of InN/InGaN quantum dots and holes and giant surface photovoltage directly measured by Kelvin probe force microscopy
Source: Sci Rep. 2020 Apr 3;10:5930. doi: 10.1038/s41598-020-62820-3 (PMC7125200; doi:10.1038/s41598-020-62820-3)
Supplement: Supplementary file 1 — Supplementary Information. [file 41598_2020_62820_MOESM1_ESM.pdf]

# Supplementary Figures

## Electric dipole of InN/InGaN quantum dots and holes and giant surface photovoltage directly measured by Kelvin probe force microscopy

Yinping Qian[1], Peng Wang[1,2], Lujia Rao[1], Changkun Song[1], Hongjie Yin[1], Xingyu Wang[1], Guofu Zhou[1,2,3]* & Richard Nötzel[1,2]*

[1]*Guangdong Provincial Key Laboratory of Optical Information Materials and Technology, South China Academy of Advanced Optoelectronics, South China Normal University, Guangzhou 510006, P. R. China.*

[2]*National Center for International Research on Green Optoelectronics, South China Normal University, Guangzhou 510006, P. R. China.*

[3]*Academy of Shenzhen Guohua Optoelectronics, Shenzhen 518110, P. R. China.*

*Corresponding authors: richard.noetzel@scnu.edu.cn, guofu.zhou@m.scnu.edu.cn.

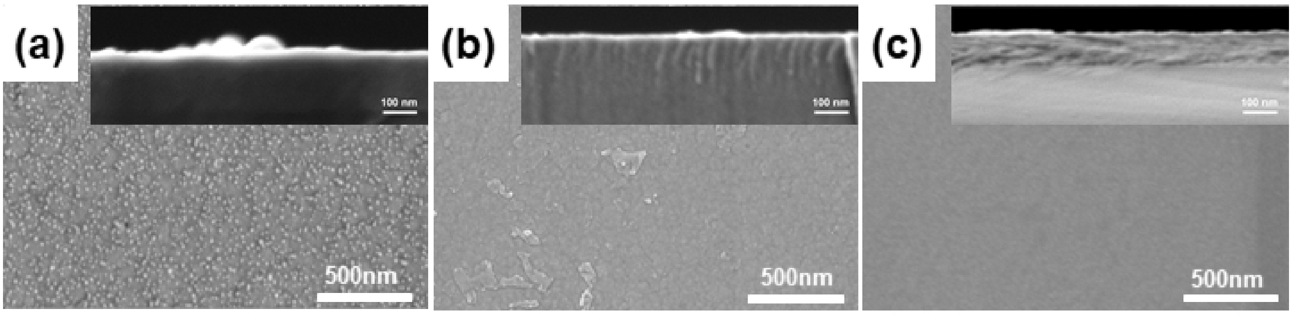


Figure S1. SEM images of the surface morphology of the as-grown (a) 1.2-ML-InN/$In_{0.45}Ga_{0.55}N$ QD structure, (b) 0.8-ML-InN/$In_{0.45}Ga_{0.55}N$ structure and (c) 1.5-ML-InN/$In_{0.75}Ga_{0.25}N$ QD structure. Insets: Corresponding cross-sectional SEM images.

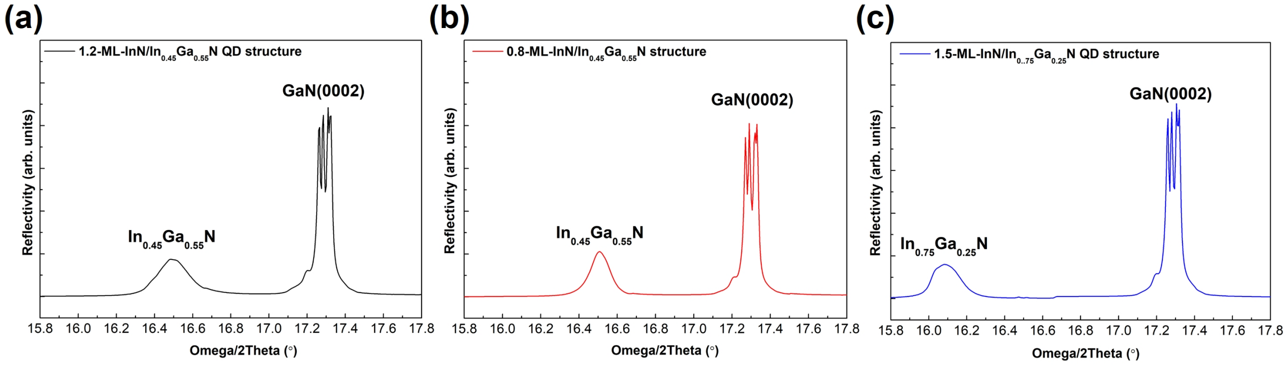


Figure S2. XRD spectra recorded around the symmetric GaN (0002) Bragg reflection using the Cu K-alpha 1 and 2 radiations of the (a) 1.2-ML-InN/$In_{0.45}Ga_{0.55}N$ QD structure, (b) 0.8-ML-InN/ $In_{0.45}Ga_{0.55}N$ structure and (c) 1.5-ML-InN/$In_{0.75}Ga_{0.25}N$ QD structure.

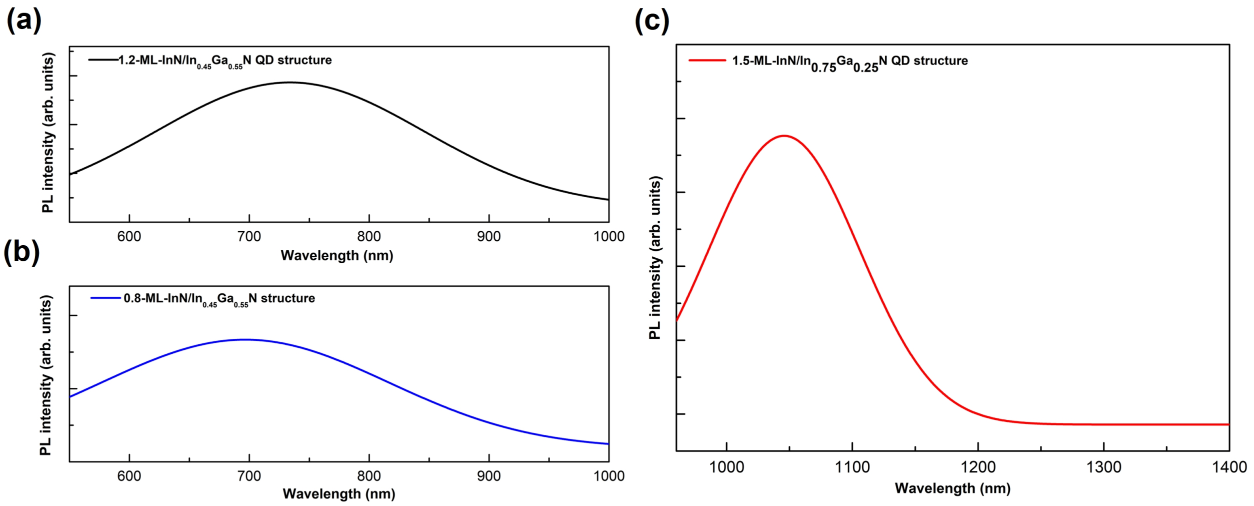


Figure S3. PL spectra at room temperature of the (a) 1.2-ML-InN/$In_{0.45}Ga_{0.55}N$ QD structure, (b) 0.8-ML-InN/$In_{0.45}Ga_{0.55}N$ structure and (c) 1.5-ML-InN/$In_{0.75}Ga_{0.25}N$ QD structure. The PL is excited by the 532 nm line of a Nd-YAG solid-state laser with excitation power of 100 mW, dispersed by a single monochromator and detected by either a Si CCD or an InGaAs single channel detector.

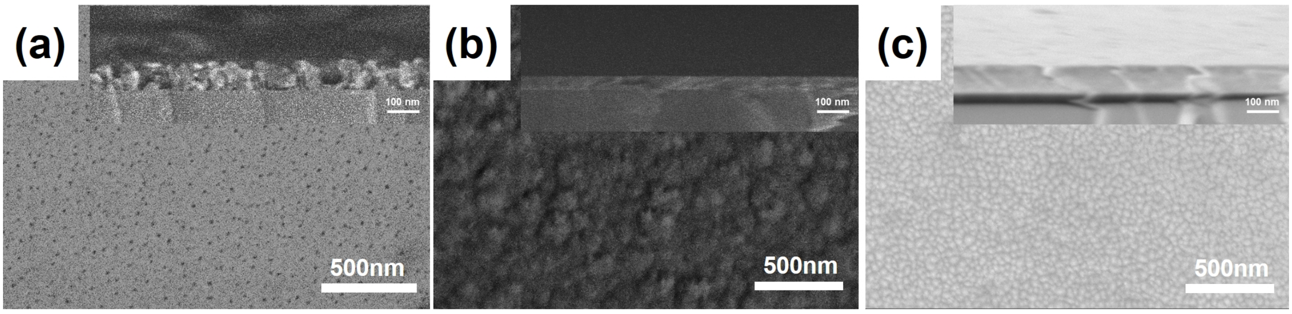


Figure S4. SEM images of the surface morphology after HCl-etching of the (a) 1.2-ML-InN/$In_{0.45}Ga_{0.55}N$ QD structure, (b) 0.8-ML-InN/$In_{0.45}Ga_{0.55}N$ structure and (c) 1.5-ML-InN/$In_{0.75}Ga_{0.25}N$ QD structure. Insets: Corresponding cross-sectional SEM images.

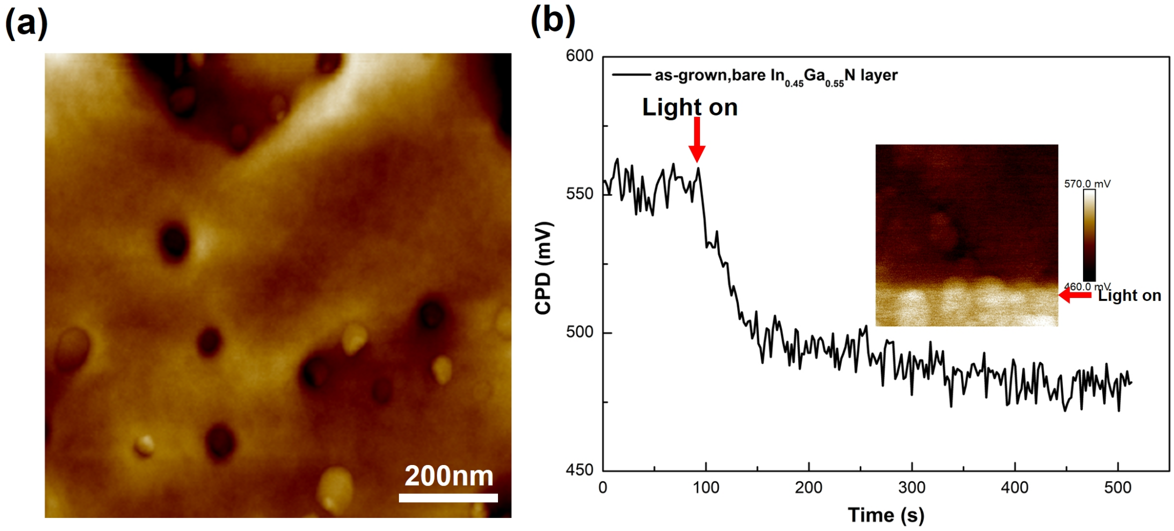


Figure S5. (a) AFM height image and (b) CPD as a function of time of the as-grown, bare $In_{0.45}Ga_{0.55}N$ layer. Inset in (b): KPFM CPD image. The arrows indicate the switching on of the light. The scan field is 1 x 1 $\mu m^2$ and the full height contrast is 15 nm in the AFM image.

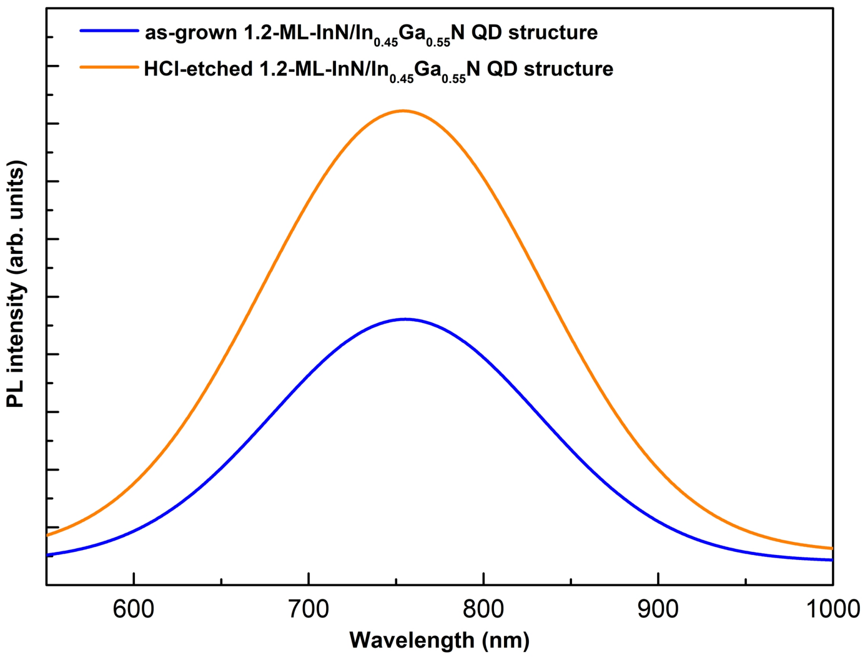


Figure S6. PL spectra at room temperature of the as-grown (blue line) and HCl-etched (orange line) 1.2-ML-InN/$In_{0.45}Ga_{0.55}N$ QD structures.

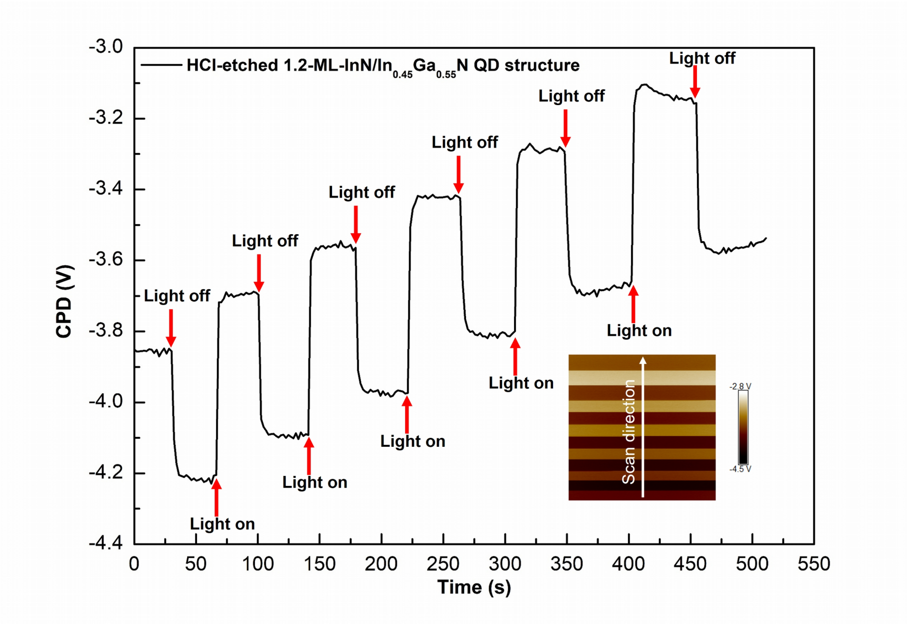


Figure S7. CPD as a function of time for the 1.2-ML-InN/$In_{0.45}Ga_{0.55}N$ QD structure after HCl-etching. The light is alternately switched on and off, as indicated. Inset: KPFM CPD image.
